# Calculation of iris-claw IOL power for correction of late in-the-bag IOL complex dislocation

**DOI:** 10.1186/s12886-017-0516-1

**Published:** 2017-07-11

**Authors:** Valentín Huerva, Francisco J. Ascaso, Isabel Caral, Andrzej Grzybowski

**Affiliations:** 1 Department of Ophthalmology, Universitary Hospital Arnau de Vilanova, Avda. Alcade Rovira Roure 80, 25198 Lleida, Spain; 2 IRB Lleida, Lleida, Spain; 3 Department of Ophthalmology, Hospital Clínico Universitario “Lozano Blesa”, Zaragoza, Spain; 4 Instituto de Investigación Sanitaria de Aragón (IIS Aragón), Zaragoza, Spain; 5 Department of Ophthalmology, Poznań City Hospital, Poznań, Poland; 6 University of Warmia and Mazury, Olsztyn, Poland

**Keywords:** IOL-in-the bag dislocation, Iris-claw IOL, Late cataract surgery complication, Artisan aphakia, Verysise

## Abstract

**Background:**

To assess the constants and formula for aphakia correction with iris-claw IOLs to achieve the best refractive status in cases of late in-the-bag IOL complex dislocation.

**Methods:**

A literature search was performed. The following data were obtained: Iris-claw IOL model, Iridal or retroiridal enclavation, A-constant, ultrasound or optical biometry, formula employed and refractive outcomes. Acceptable emmetropia was considered if the resulting spherical equivalent (SE) was within ±1.00 D.

**Results:**

The majority of the studies used SRK/T formula (66.6%). The 88.9% of the reports obtained a SE within ±1.00 D. Using A-115 for ultrasound biometry and A-115.7 for optical biometry and SRK/T formula, the emmetropia (±1.00 D) of SE, was able to get near 100% of reported cases over the pupil implantation. However, the emmetropia decreased to 80% when the enclavation is retropupilar using the same formula. The A-constant can vary from 116.7 to 117.5 for retropupilar enclavation.

**Conclusions:**

Using A-115 for ultrasound biometry and A-115.7 for optical biometry and SRK/T formula, ±1.00 D of SE, is able to get near 100% of cases. Nevertheless, ±1.00 D of SE decreased to 80% of the cases when the enclavation is retropupilar.

## Background

Decentration or luxation of a posterior chamber intraocular lens (IOL) is an uncommon problem following cataract surgery. The phenomenon late in-the-bag IOL complex dislocation after an uneventful cataract surgery can occur many years postoperatively, due to previous progressive zonular disintegration and capsular shrinkage [1–6].

Pseudoexfoliation syndrome is present in more than 50% of the reported cases [6]. Other risk factors such as advanced age, high myopia, uveitis, trauma, previous vitreoretinal surgery, diabetes mellitus and connective tissue disorders may be also associated [6]. Complications during cataract surgery and advanced status of the cataract also increased the risk of late in-the-bag IOL dislocation [7]. No significant differences have been found following extracapsular cataract extraction when compared with phacoemulsification [8]. However, a long phacoemulsification time may be also a risk factor [9]. Although, the cumulative risk of IOL dislocation remains low after cataract surgery, an increased occurrence of late in-the-bag IOL dislocation can be expected in postoperative years due to life expectancy [5]. On the other hand, high myopia may be the main risk factor in other studies [10]. Many myopic refractive procedures have been performed by means of clear lens extraction. After a 10-years follow-up a 0.6% of cases may require a surgical procedure for a dislocated IOL [11]. Until now, it is not clear if this percentage could increase in cases operated of myopia in future years.

The mean time after cataract extraction and a secondary surgery for late in-the-bag IOL dislocation is approximately 8.5 years [12–14]. When in-the-bag IOL dislocation occurs, due to the risk of further decentration, it is usually better to perform surgery while an anterior approach is still possible. Iris-claw lens insertion has been shown their utility in acquired aphakia conditions in the overwhelming majority of cases, with little evidence of post-operative problems such as uveitis, glaucoma, or hyphema [15–17]. The exchange of the dislocated IOL-bag complex and implantation of an iris-claw IOL for aphakia correction during the same time may be a safe and predictable technique with minimal complications [18]. Many reports with iris-claw IOL implantation for correction of this syndrome have been reported. Nevertheless, there is no consensus about the A-constant and the employed formula for determining IOL power. In the majority of studies, the authors do not mention about A constant and employed formula [19]. This fact constitutes a challenge for achieving an emmetropia when the extraction and implantation of an iris-claw IOL would be at the same time.

The aim of the study is to assess readers in the best A-constant and formula employed in different studies reporting aphakia correction with iris-claw IOLs to achieve the best refractive status following surgery in cases of late in-the-bag IOL complex dislocation.

## Methods

A literature search was performed using Pubmed and Google Scholar until February 2017. The keywords used were: late in-the-bag IOL dislocation, IOL luxation, cataract and pseudoexfoliation syndrome, iris-claw IOL and aphakia, Artisan aphakia, Verisyse aphakia. References cited in the identified reports were also reviewed. Only the reports that have reference to iris-claw IOL implantation in cases of in-the-bag IOL dislocation in the same time were considered. From the articles identified, the following data were obtained if were referenced: Iris-claw IOL model, Iridal (over the pupil) or retroiridal (retropupillary) enclavation, A-constant, ultrasound or optical biometry, formula employed and refractive outcomes. Reports that do not refer the majority above mentioned data in the material and methods were discarded. From the reports that have reference to this situation only the cases operated in the same time dislocated complex IOL bag extraction and implantation of iris-claw in the same time were choosen for the study. Acceptable emmetropia was considered if the resulting spherical equivalent (SE) was within ±1.00 D. The A-constant provided by the manufacturer of the iris-claw IOLs (Opthec, 9700 AJ Groningen, The Netherlands) were also consulted [20].

## Results

### Data provided by the manufacturer

Regarding refractive iris-claw IOLs in phakic conditions keratometry and anterior chamber depth (ACD) were taken into account for the power IOL calculation by means of a calculator [20]. The van der Heijde formula is usually employed in phakic conditions [21]. van der Heijde formula is a theoretical formula of first generation. The problem with this formula is the need to predict from the preoperative data the position that the IOL will take within the eye in a pseudophakic eye. Do not confuse pseudophakic anterior chamber depth (ACD) with preoperative phakic ACD.

The A-constant provided by the manufacturer is 115.0 (ultrasound) and 115.7 (optical biometry) for Artisan® aphakia IOL (model 205, Ophtec, Groeningen, The Neederlands) for the SRK/T formula. There are available powers from +2.00 to +30.00 D [22]. Verisyse® IOL for aphakia model VRS54 from Abott (Abott Laboratories, Santa Clara, US) is the same IOL that Artisan® aphakia model 205 from Ophtec aproved by the FDA in United States [23]. The A-constant is the same. Refractive models for myopia correction may be employed in special situations because there is not a power lower than +2.00 D for Artisan® aphakia IOL [24].

### Data of the revised manuscripts

Many reports treat the beneficial outcomes after an iris-claw IOL implantation to resolve the aphakia. However, there are few reports that have reference to the employed biometry, constant and final spherical outcomes after dislocated IOL-bag extraction and iris-claw implantation. Many consulted studies did not report the A-constant and formula employed and, therefore, they were discarded. Table 1 summarizes the eight reviewed reports with the number of cases relating the procedure in the same time and refractive outcomes after the iris-claw IOL enclavement in this situation. Many of them report the iris-claw IOLs implant in other situations.

**Table 1:** Outcomes after using Iris-claw IOLs for aphakia correction. SE: Spherical Equivalent. NR: Not referenced

Author	N° cases. Iris-claw model	Iridal (over the pupil)/retroiridal enclavement	A-constant employed	Ultrasound /Optical biometry	Formula employed	Refractive Outcomes
Da Silva et al. [16]	18. Artisan/Verisyse Aphakia	Iridal	115.0	NR	NR	SE: +0.12 ± 1,76 (−2 D to +1.62D)
Huerva et al. [18]	3. Artisan Aphakia	Iridal	115.7	Optical	SRK/T	SE (−0.75 D to +0.50D) past 1 year
Huerva [23]	1. Artisan Myopia	Iridal	103.8	Optical	Modified SRK/T (T2 Formula) [29]	SE: +0.125 D
Gonnermann et al. [31]	95. Artisan/Verisyse Aphakia	Retroiridal	116.9	NR	SRK/T	SE: ± 1D in 75.9% of cases
Farrahi et al. [30]	12. Artisan Aphakia	Iridal	NR	Ultrasound	SRK/T	SE: mean 0,06D (range: −1,75 to 2,5)
Wolter-Roessler et al. [29]	16. Artisan	Retroiridal	116.7	NR	SRKT	SE: 0 (−0.50 to +1D
Schmidt et al. [26]	17. Verisyse	Reroiriridal	SRKT:116.8 SF:0.8, pACD:4,4 and a0:0,49 for the Haigis formula.	NR	SRKT.Holaday, HoferQ and Haigis	SE: +/−2 D
Choragiewicz et al. [27]	27. Artisan Verisyse	Retroiridal	A0: −0,25, A1: 0.4, A2: 0,1	NR	Haigis	SE: - -0.27 D (−3.87 to +2.8)

There are articles describing isolated case reports in special situations as high myopia [24]. Most of reports describe the implantation of an Artisan® aphakia 205 iris claw IOL or Verisyse® iris claw IOL.. From the selected studies the majority (66.6%) used SRK/T formula. Only one of them modified the formula for a specific case associated to high myopia using the T2 formula [24, 25]. The Haigis formula was used in two reports [26, 27]. Holladay 2 and Hoffer Q were referenced only in a report [26]. In cases over the pupil implantation, the A-constant employed is 115 for presumably ultrasound biometry [16] and 115.7 for optical biometry [18]. A-103.8 was used when an Artisan myopia® model was used to correct the aphakic situation [24]. A-constant for retropupilary enclavation with SRK/T formula varies from 116.7 to 117.5 [24, 26, 28, 29]. A0 constant used for Haigis formula differ in two reports [26, 27]. The 88.9% of the reports obtained a SE within ±1.00 D [16, 18, 24–27, 29]. In the 44.4% the enclavation was over the pupil and generally the SRK/T formula was employed [16, 18, 24, 30]. In the 55.6% of reports the enclavation was retroiridal [23, 26–29, 31] and the SRK/T formula was employed in the 80% of the reports [23, 26, 28, 29]. With this formula almost 60% of cases could achieved the emmetropia within ±1.00 D. However, differences on the A-constant employed are notable between all reports. A-116.9 for optical biometry may be the mean for the SRK/T formula in large reported series that employer retroiridal enclavement [23, 32].

## Discussion

Surgical correction of the aphakia results in a challenging decision and approach. One solution may be the reposition and suture of the IOL to the scleral wall [33]. The advantage of repositioning and suturing the IOL is that it may avoid a large incision and astigmatism induction. Nevertheless, in some cases this technique is not possible because either IOL is dislocated into the vitreous cavity or vitreous appears into the anterior chamber. An exchange of the IOL-bag complex may be preferable in these situations. Angle supported IOLs or iris-claw IOLs may resolve the aphakia at the same time of surgery or in a second time. However, anterior chamber angle supported IOLs are not popular because they may produce corneal decompensation in compromised corneas [34]. Another possibility is the scleral-fixated PC-IOLs, but they may produce lens tilting and decentration among others [34–36].

There are few reports about treatment of aphakia in the late in-the-bag dislocation with an iris claw IOL. Description about the formula and A-constant employed are summarized in Table 1. Retroiridal enclavation is reported more frequently in the last time. The theoretical advantage of this location is to avoid the endothelial cell loss [37]. However, the largest series reported show no significant endothelial cells loss after over the pupil enclavation [38, 39]. A study showed that there were no differences in endothelial cells count between three techniques for aphakia correction: anterior fixated iris claw, posterior iris fixated IOL and scleral-fixated IOL [40]. The percentage of endothelial cells loss can vary in the different studies between 5.5 and 11.7% according to their follow-up [37, 41]. The majority conclude that the major loss occurs during the first year, due to the surgical trauma during the implantation as it occurs with the implantation of phakic iris claw IOLs [42]. According to these results, anterior or posterior enclavation of the iris claw depend of the decision, ability and preference of the surgeon.

Most authors report the implantation of an iris claw IOL to resolve the refractive status after aphakia, trauma, vitrectomy or complicated cataract surgery. However, an iris-claw IOL may be implanted at the same time of the extraction of the dislocated IOL-bag complex. Because of this, it is necessary to observe the reported refractive outcomes with the use of iris-caw IOLs for aphakia.

We observe eight studies reporting cases with explantation of the dislocated IOL-bag complex and implantation of an iris-claw at the same time [16, 18, 23, 24, 26–30]. Since many refractive procedures have been performed by means of clear lens extraction and IOL implantation in myopic population, it is probable in a higher frequency of late in-the-bag IOL dislocation cases in the future. The duration of surgery and pseudoexfoliation syndrome may increase the possibility of this syndrome about 8.5 years later [9–11, 43, 44]. In this sense, it is possible to perform the extraction of the dislocated complex and implantation of an iris claw IOL due the above mentioned advantages and get close to emmetropia.

Emmetropia can be achieved within ±1.00 D of SE near of 100% of cases using the SRK/T formula in cases over the pupil enclavement using the A-constants of the manufacturer [16, 18, 24, 30] and also near 80% of cases with the same formula for posterior enclavement. Nevertheless, for posterior enclavation there are no clear consensus for A-constant employed in this study [23, 26, 28, 29]. Although, with an A-constant of 116.9 the emmetropia can be achieved within ±1.00 D of SE in other study about retroiridal enclavation of an iris-claw IOL [32]. For the cases out of ±1.00 D of SE the corneal curvature and axial length should be considered and optimized constants for SRK/T, Haigis, Hoffer Q, and Holladay 1 formulas as for primary IOL implantation during cataract surgery or refractive lens exchange should be necessary to minimize the deviation between postoperative refraction and the target refraction. The revised studies have employed mostly the SRK/T. An alternative may be the Holladay 2 formula used in a study with megalocornea [45] or modification of SRK/T employed in another one in cases of high myopia [23]. The great SE deviation is observed in the report that use all formula [26]. When the Haigis formula is used emmetropia can be achieved; however, a great standard deviation is observed [27].

## Conclusions

The iris-claw IOL implantation may be a safe and predictable method to correct the aphakia at the same time in cases of late in-the-bag IOL dislocation. Better standardized constants and the possibility of 4th formula generation (Olsen, Barrett, Hill, etc.) should be considered to obtain the emmetropia. This would be especially necessary in cases of retroiridal implantation. Currently, there is no literature on the latest formulas and the use of iris-claw IOLs.

## Abbreviations

ACD: Anterior chamber depth
D: Diopter
FDA: Food and drug administration
IOL: Intraocular lens
PC-IOLs: Posterior chamber intraocular lenses
SE: Spherical equivalent
